# Timely rehabilitation for critical patients with COVID-19: another issue should not be ignored

**DOI:** 10.1186/s13054-020-02967-7

**Published:** 2020-06-02

**Authors:** Zhen Wang, Zhongjing Wang, Ruixiang Sun, Xiaoye Wang, Shaofei Gu, Xiancui Zhang, Houbao Huang

**Affiliations:** 1 The 6th Group of Anhui Medical Assistance Team for Wuhan, Wuhan, China; 2 Department of Critical Care Medicine, Yijishan Hospital, First Affiliated Hospital of Wannan Medical College, No. 2 Zheshan West Road, Wuhu, Anhui China; 3 Department of Endocrinology, The Central Hospital of Wuhan, Tongji Medical College, Huazhong University of Science and Technology, No.14 Gusaoshu Road, Wuhan, Hubei China; 4 Department of Nursing, Yijishan Hospital, First Affiliated Hospital of Wannan Medical College, No. 2 Zheshan West Road, Wuhu, Anhui China; 5 Department of Traditional Medicine, Yijishan Hospital, First Affiliated Hospital of Wannan Medical College, No. 2 Zheshan West Road, Wuhu, Anhui China; 6 Department of Urology, Yijishan Hospital, First Affiliated Hospital of Wannan Medical College, No. 2 Zheshan West Road, Wuhu, Anhui China

Dear Editor,

We have read with a passionate interest in the manuscript of Li et al., which revealed some essential concerns that critical care medicine should learn during of the coronavirus disease-19 (COVID-19) pandemic [1]. Amongst their suggestions, the authors underlined that intensivists should extensively cooperate with multiple disciplines to respond to this public health crisis.

Nonetheless, similar to the other voluminous dissertations focused on the treatment of severe COVID-19, Li et al. regrettably neglected the field of the rehabilitation of critically ill patients. However, growing evidence has indicated that critical illness often leads to the impairment of physical, mental, psychological, and social function in the survivors [2, 3].

Among the 41 patients confirmed with COVID-19 that our team cured in the central hospital of Wuhan, there were 14 severe cases and four critical cases. The Medical Research Council Scales in 65.8% of patients were lower than 48 and lower than 35 in severe and critical individuals. Meanwhile, 93% of patients displayed varying degrees of anxiety, 96% of patients fell into a sleep disorder, and more than 90% of patients exhibited panic attacks of the disease uncertainties (detailed unpublished data).

To triumph over these secondary damages from COVID-19, we have developed one generalized rehabilitation strategy characterized as a hierarchy, upgrade, and integration of traditional and western medicine. One multidisciplinary team performs rehabilitation treatment. First, physicians of western medicine and traditional Chinese medicine collectively assess the clinical situation of the patient. Second, the physical therapists and psychiatric nurses evaluate the physical and mental function, respectively. Third, there should be a panel meeting to decide the timely rehabilitation scheme that includes physiotherapy interventions and psychological counseling.

There are some successful experiences of overcoming the shortage of professional resources in the isolation ward. Heightening the health workers’ comprehension of the rehabilitation facilitates early recognition of asthenia or mental dysfunction. Fast popularization of the physiotherapy skills among the nurses could lighten the workload of physiotherapists. Using social applications such as WeChat to connect the patients, families with medical staff play a role in psychological intervention. Moreover, Tai Chi Chuan and Baduanjin, two kinds of mind-body exercise origin from Chinese traditional medicine, characterized as easy-learning, improvements in motion, strength, emotional, and social attributes, are worth promoting in selective patients [4].

Based on our experiences, the tissue of timely rehabilitation for these patients should not be ignored at the moment of the continuous COVID-19 epidemic worldwide.

## Authors’ response

Li Li, Jing Yan

Dear Editor,

Wang et al. mentioned that timely rehabilitation cannot be ignored for critical patients, which is enlightening. We have also noticed that early active rehabilitation can help the recovery of cardiopulmonary function and physical fitness and reduce the psychological pressure of critically ill patients. Furthermore, Shen et al. mentioned that not only patients but also the medical staff is under enormous psychological pressure [5].

Nonetheless, there are additional issues posed in the ICU. The core of this article is an opinion on the ten critical issues for ICU that needs to be strengthened when facing a major public health disaster [1], which is only an urgent and core opinion. Some measures, such as strengthening occupational protection training and developing special standardized protection procedures, are challenging and have significant risks in many countries. For example, in Spain, more than 40,000 personnel, i.e., about 20% of the medical staff is infected with COVID-19, increasing the patient’s treatment resources. We also mentioned the specific disease database and specimen bank and shared the research data. China has established a valuable database for patients from the beginning, continuously published a large number of scientific articles, summarized clinical experience, and provided valuable clues and information to combat the epidemic at the global level. We also mentioned the need for multi-disciplinary cooperation, including infectious diseases, respiratory, nephrology, intensive care, and pathology physicians. Also, the participation of rehabilitation physicians and psychologists, mentioned by Wang et al., is crucial.

In addition to the ten critical issues we mentioned, several other problems need to be addressed in response to the prevalence of COVID-19 [6]. Especially as the epidemic intensifies, and even from an emergency, public health event to a normalized infection, intensive care medicine will face new challenges. If SARS-CoV-2 continues to infect humans and the vaccine has not been successfully developed, the workload of critical care physicians and the resources of medical staff would be urgent issues that need a resolution. Moreover, a large number of patients with COVID-19 may also exhibit many primary diseases, and hence, critical care physicians need more support and cooperation from other professional discipline teams for the treatment of the patients and restore their health.

Furthermore, COVID-19 has forced the shift in the allocation of medical resources and delayed the treatment of other diseases, such as tumors, cardiovascular diseases, and diabetes. Thus, these new issues should be worthy of our attention and consideration.

## References

[1] Li L, Gong S, Yan J (2020). COVID-19 in China: ten critical issues for intensive care medicine. Crit Care.

[2] Bein T, Weber-Carstens S, Apfelbacher C (2018). Long-term outcome after the acute respiratory distress syndrome: different from general critical illness?. Curr Opin Crit Care.

[3] Duan L, Zhu G (2020). Psychological interventions for people affected by the COVID-19 epidemic. Lancet Psychiatry.

[4] Tao J, Liu J, Liu W, Huang J, Xue X, Chen X, Wu J, Zheng G, Chen B, Li M (2017). Tai Chi Chuan and Baduanjin increase grey matter volume in older adults: a brain imaging study. J Alzheimers Dis.

[5] Shen X, Zou X, Zhong X, Yan J, Li L. Psychological stress of ICU nurses in the time of COVID-19. Crit Care. 2020. 10.1186/s13054-020-02926-2. 10.1186/s13054-020-02926-2 PMC7202793 32375848

[6] Jason P, Li W, Lowell L, et al. Intensive care management of coronavirus disease 2019 (COVID-19): challenges and recommendations. Lancet Respir Med. 2020. 10.1016/S2213-2600(20)30161-2. 10.1016/S2213-2600(20)30161-2 PMC7198848 32272080

